# Supplementary figures and images for: A new model for freedom of movement using connectomic analysis
Source: PeerJ. 2022 Aug 11;10:e13602. doi: 10.7717/peerj.13602 (PMC9375968; doi:10.7717/peerj.13602)

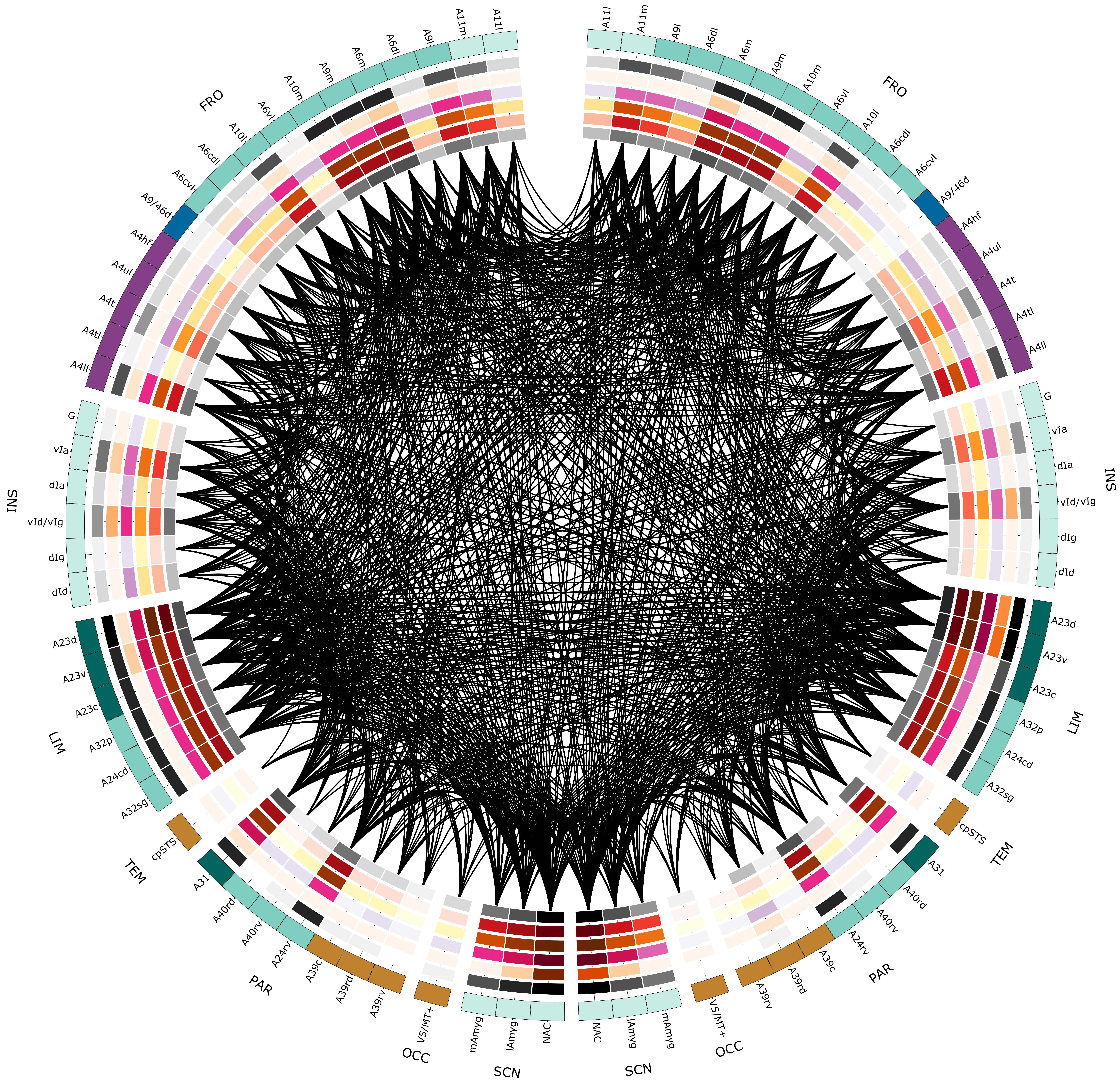

Supplement: Supplemental Information 2 — Connectivity of all 82 nodes involved in all eight movement processes is shown. Nodes are grouped by brain regions. Nodes of the left hemisphere are shown at the left of the graph, and those of the right hemisphere at the right. Inside, concentric circles represent the six centrality measures (from outermost to innermost: authorities, betweenness, degree, eigenvector, hubs and closeness centrality criteria) and their values are color-coded following a temperature graph at the bottom. These values were normalised as described in Materials & Methods. Abbreviations: FRO, frontal lobe; INS, insula; LIM, limbic system; TEM, temporal lobe; PAR, parietal lobe; OCC, occipital lobe; SCN, sub-cortical nuclei. [file peerj-10-13602-s002.pdf]

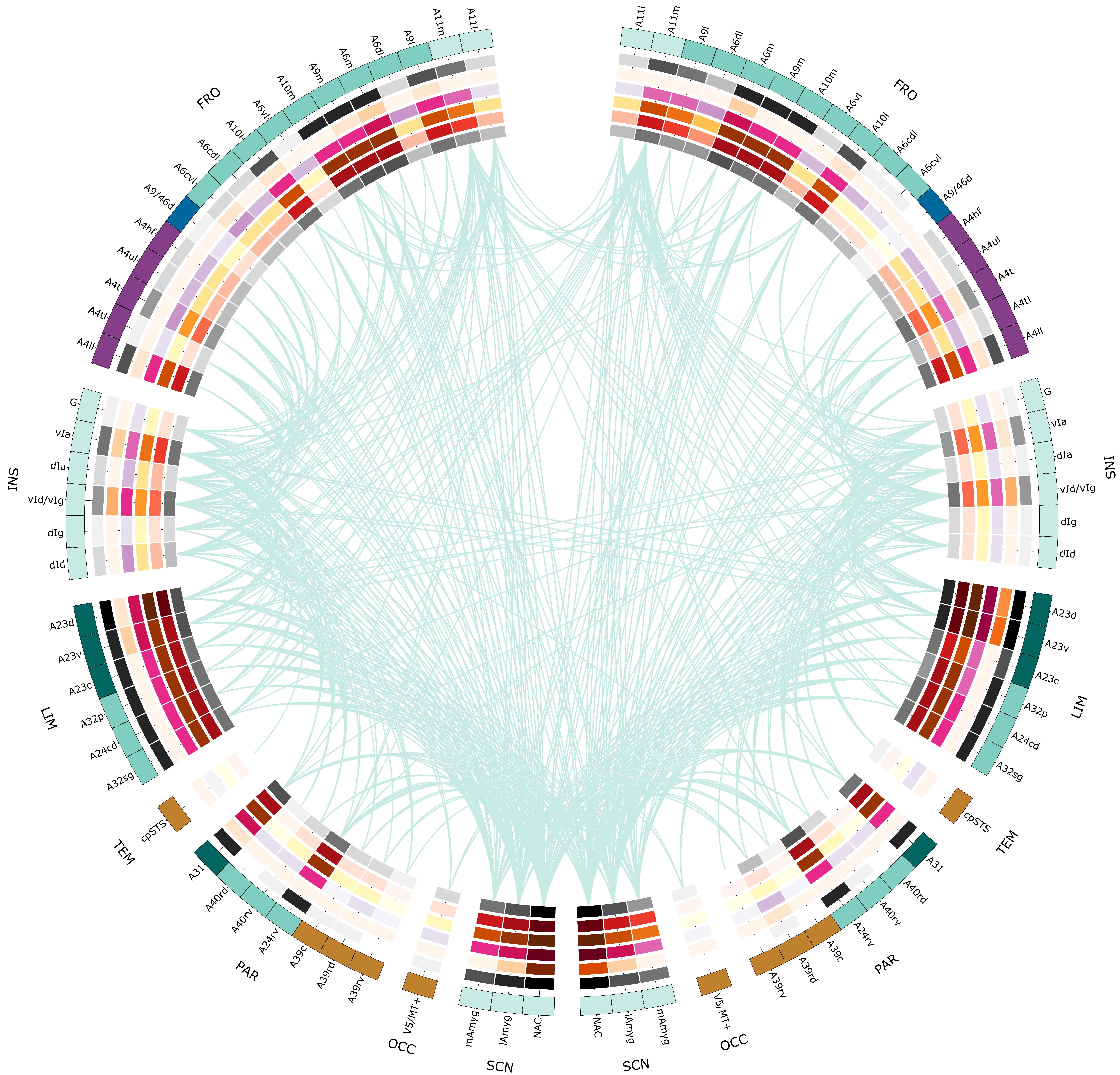

Supplement: Supplemental Information 3 — Connectogram organisation and abbreviations as in Fig. S1. Nearly all of the 82 nodes are connected to at least another node in this process. The overall connectivity pattern of sub-areas involved in Motivation is extensive and similar in both hemispheres. [file peerj-10-13602-s003.pdf]

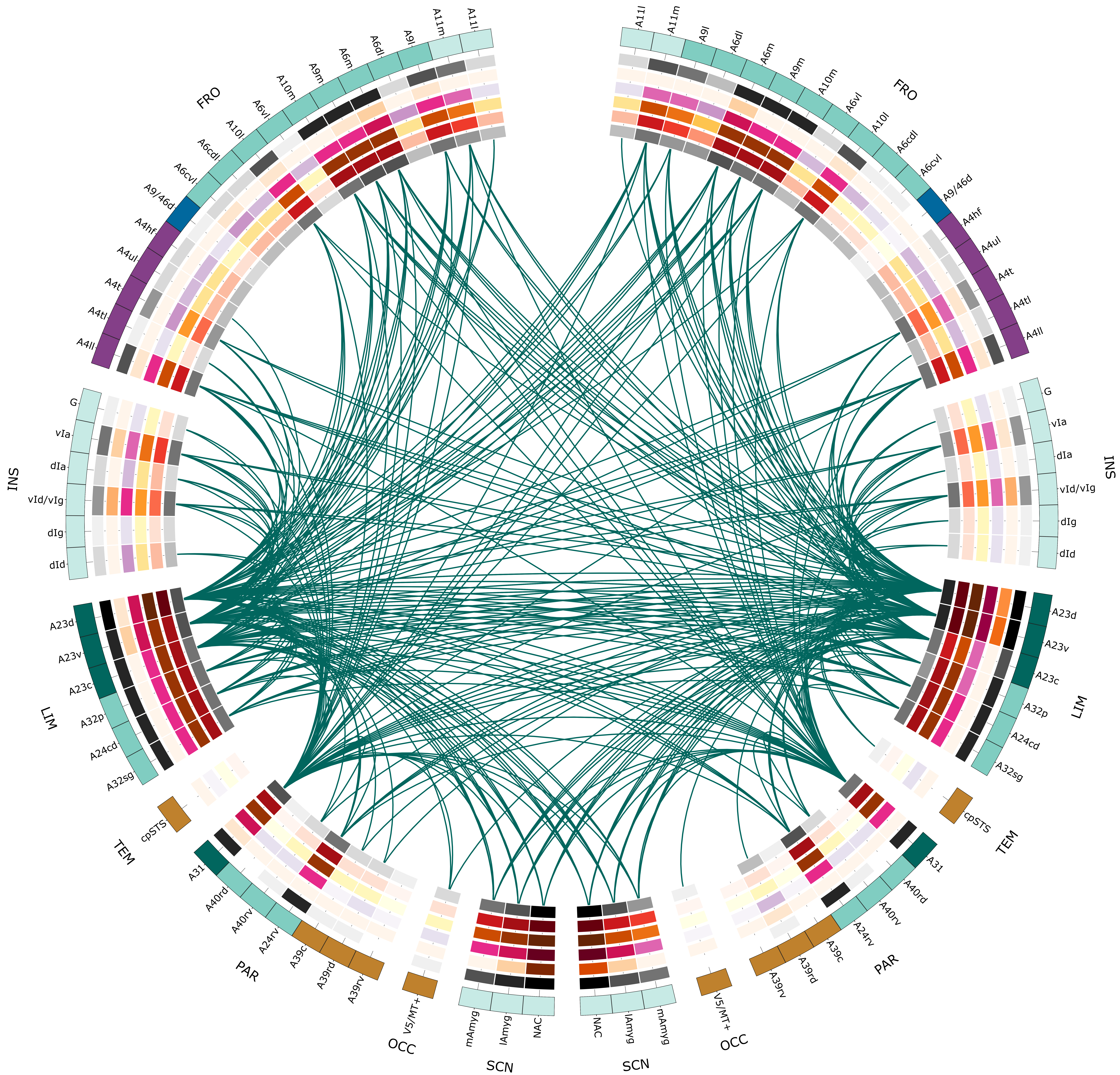

Supplement: Supplemental Information 4 — Connectogram organisation and abbreviations as in Fig. S1. Most sub-areas involved in modulation of Motivation show comparatively higher centrality values except for betweenness centrality. [file peerj-10-13602-s004.pdf]

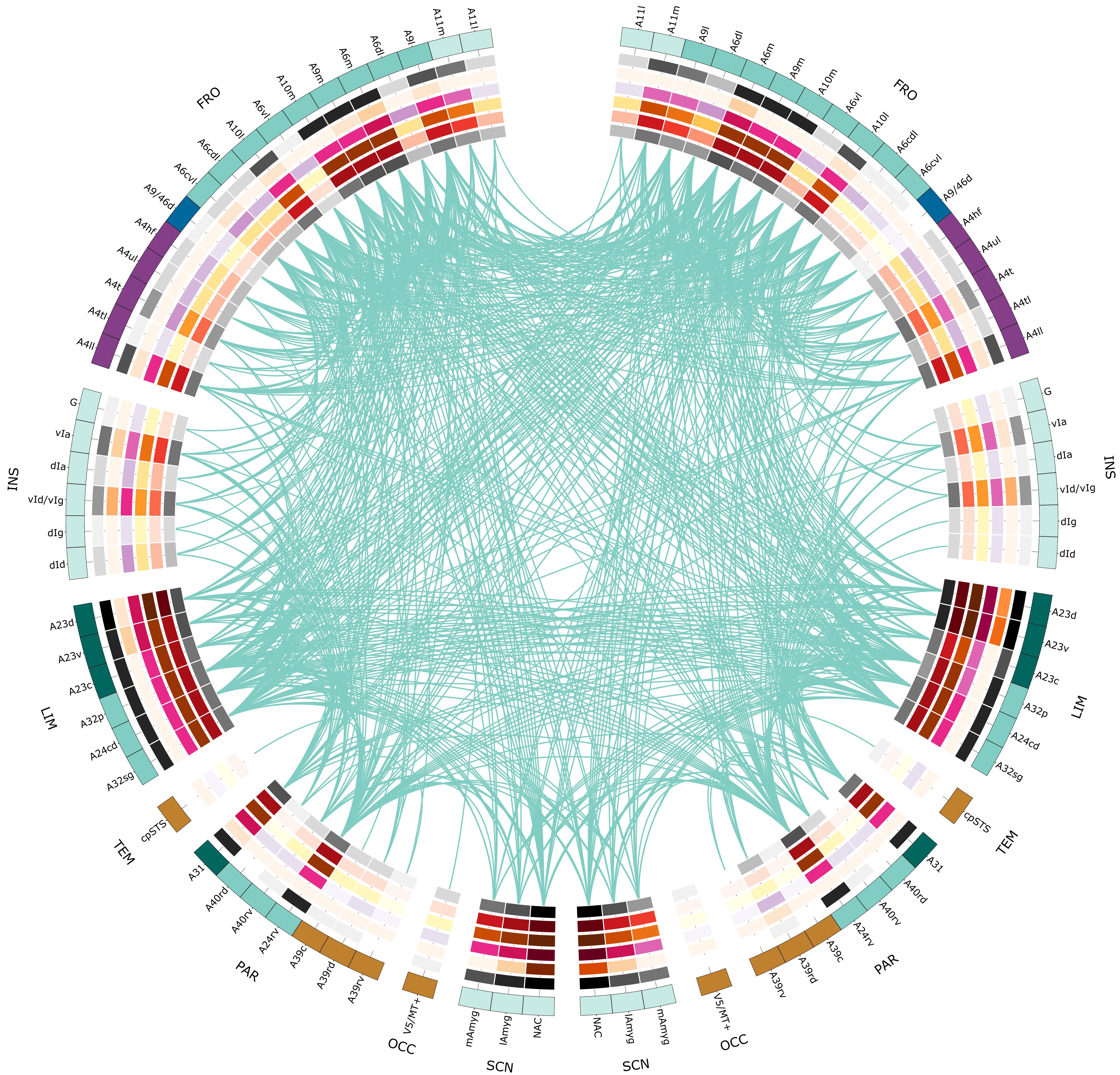

Supplement: Supplemental Information 5 — Connectogram organisation and abbreviations as in Fig. S1. Most of the 82 nodes are connected to at least another node in this process. Planning is the process that presents more connectivity in the volitional network. [file peerj-10-13602-s005.pdf]

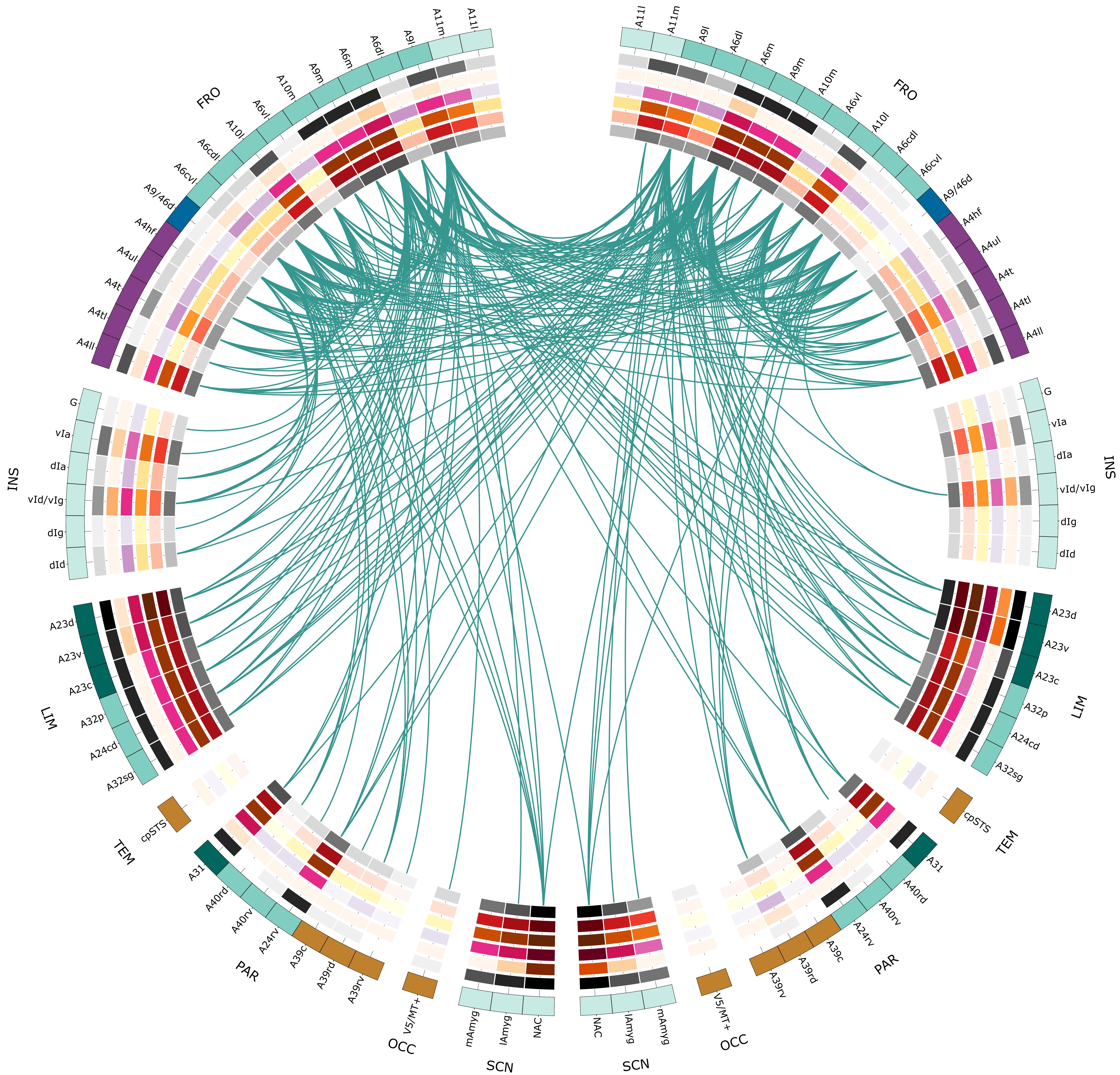

Supplement: Supplemental Information 6 — Connectogram organisation and abbreviations as in Fig. S1. Connections involved in Timing occur in the frontal lobe mostly and some bilateral differences are observed. [file peerj-10-13602-s006.pdf]

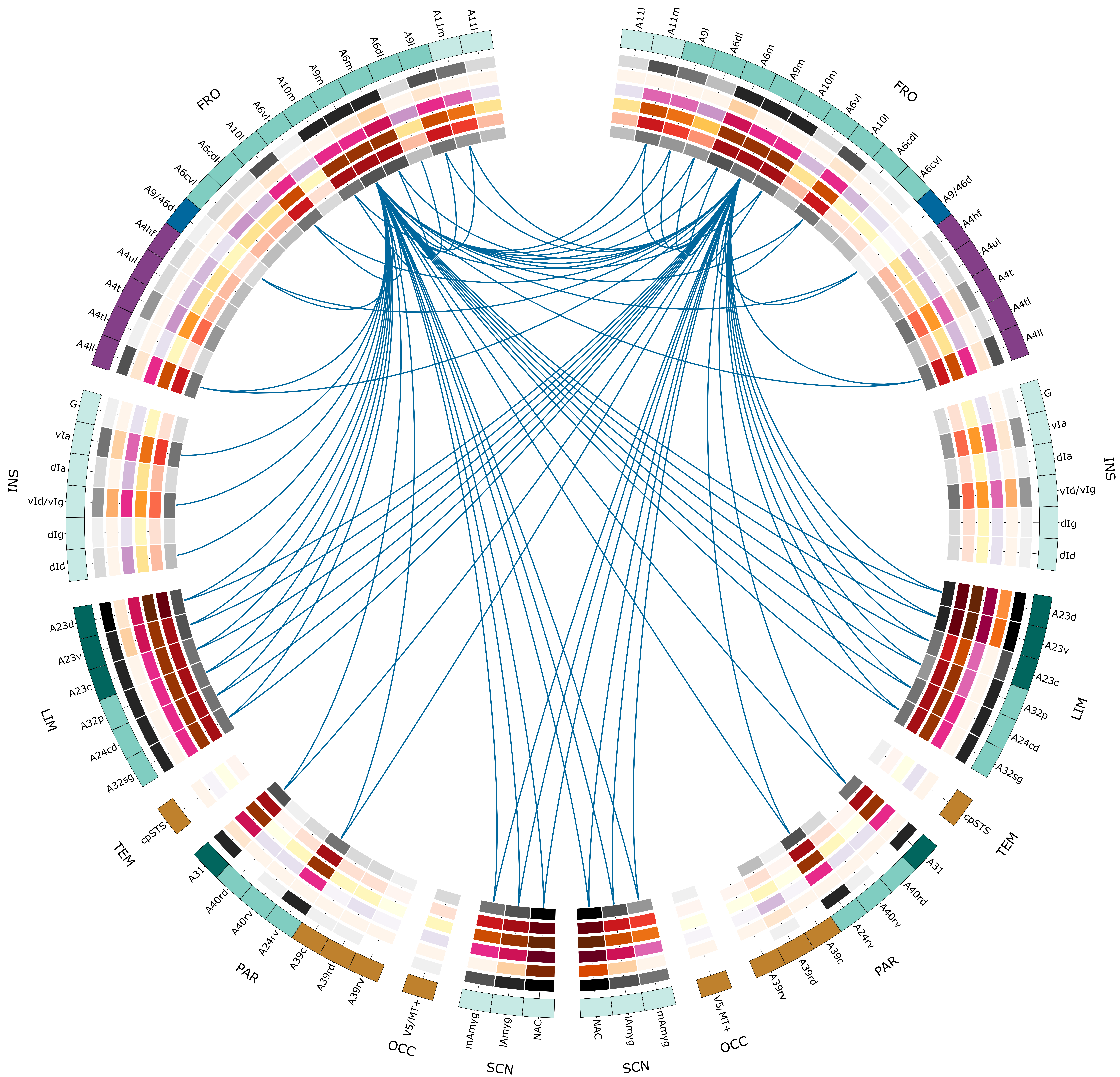

Supplement: Supplemental Information 7 — Connectogram organisation and abbreviations as in Fig. S1. Connectivity is observed mainly between A9m and many sub-areas in the frontal lobe, parietal lobe, limbic areas, and subcortical nuclei in both hemispheres, with both ipsilateral and contralateral connections. [file peerj-10-13602-s007.pdf]

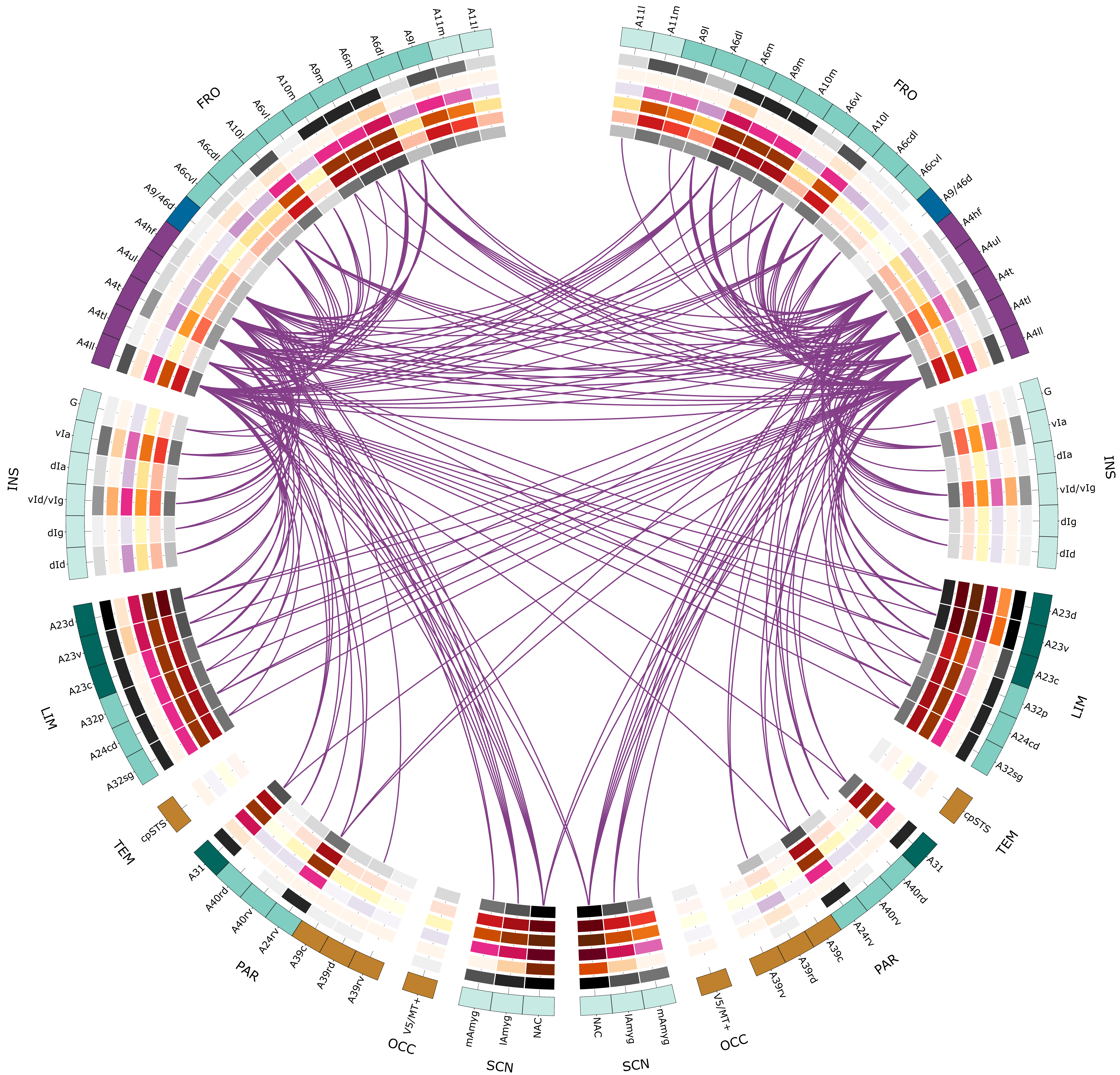

Supplement: Supplemental Information 8 — Connectogram organisation and abbreviations as in Fig. S1. Connections are observed among several sub-areas in the frontal and parietal lobes, in the insula, limbic areas and subcortical nuclei. The overall pattern of connectivity looks symmetrical and no connections are observed in the temporal and occipital lobes. [file peerj-10-13602-s008.pdf]

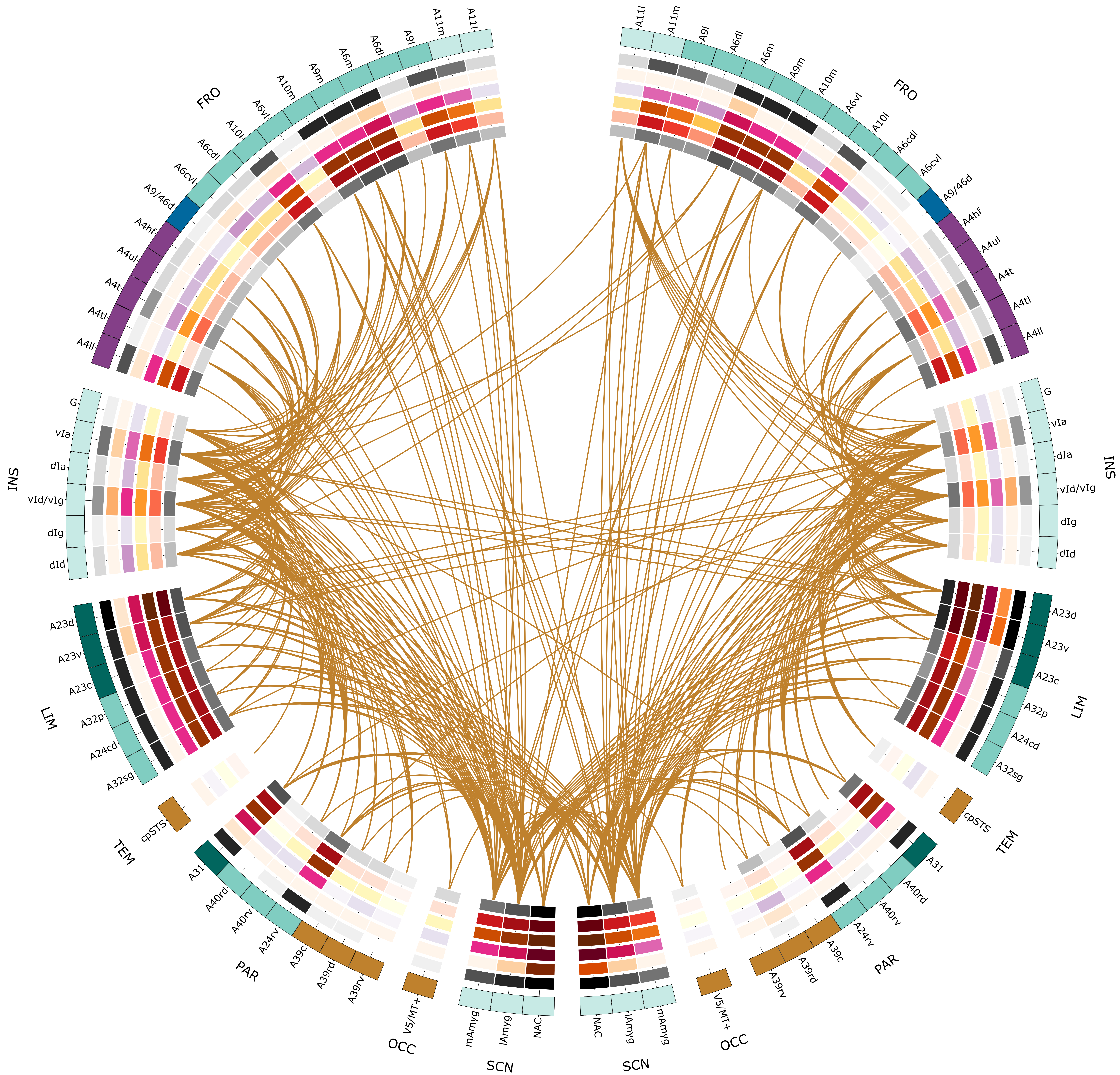

Supplement: Supplemental Information 9 — Connectogram organisation and abbreviations as in Fig. S1. Connectivity is extensive in all regions examined with a bilateral difference in the insula. [file peerj-10-13602-s009.pdf]

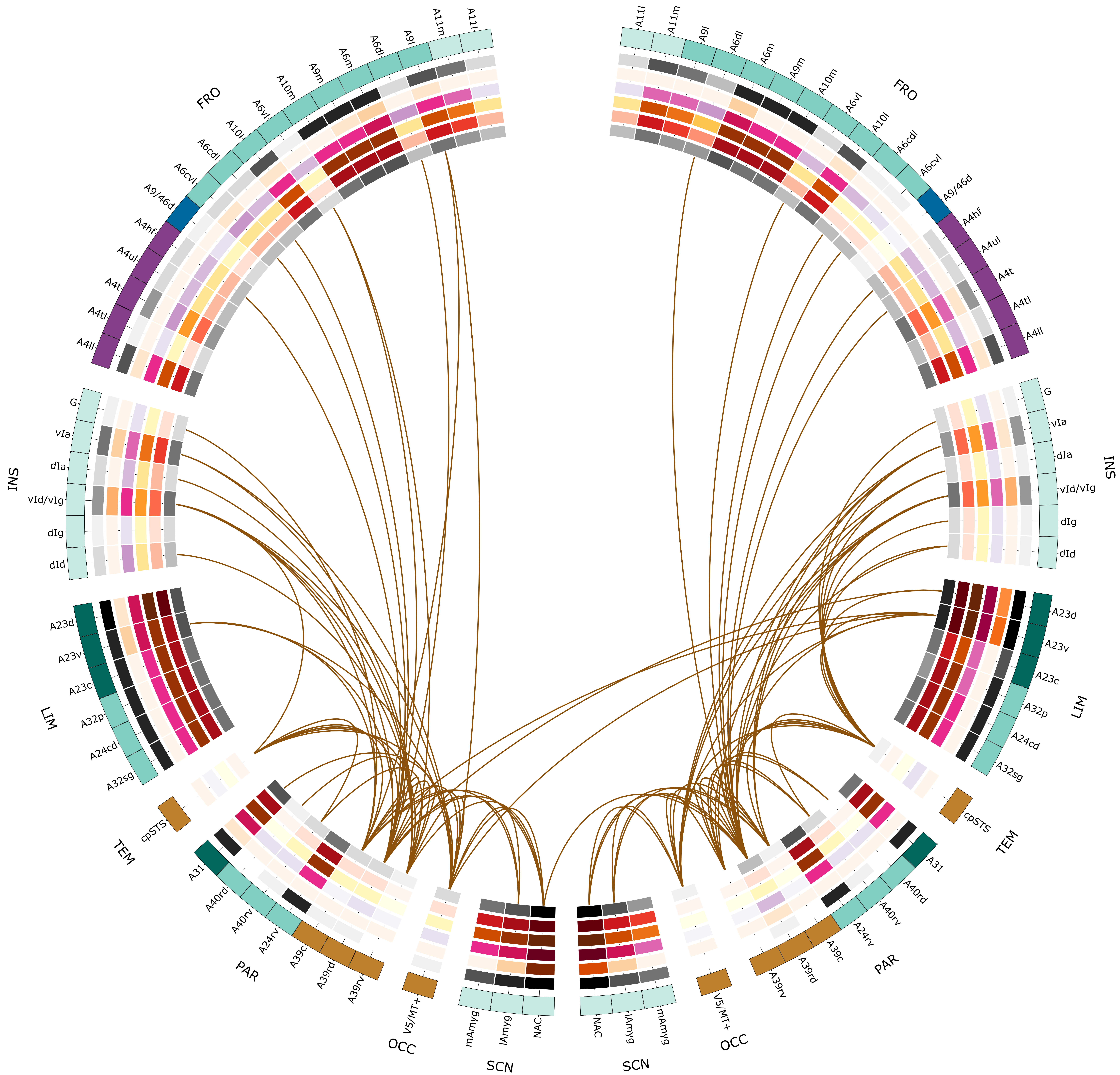

Supplement: Supplemental Information 10 — Connectogram organisation and abbreviations as in Fig. S1. A more restricted connectivity is observed with some bilateral differences. Nodes involved in modulation of Agency present the lowest centrality values. [file peerj-10-13602-s010.pdf]
